# Pancreatic Cancer Resectability After Neoadjuvant Treatment: An Imaging Challenge

**DOI:** 10.3390/diagnostics15141810

**Published:** 2025-07-18

**Authors:** Ioannis Christofilis, Charikleia Triantopoulou, Spiros Delis

**Affiliations:** 1 Radiology Department, Konstantopouleio Hospital, 142 33 Athens, Greece; 2 HPB Unit, Konstantopouleio Hospital, 142 33 Athens, Greece

**Keywords:** pancreatic cancer, neoadjuvant therapy, computed tomography, resectability, vascular involvement, imaging biomarkers

## Abstract

**Background:** Assessing pancreatic ductal adenocarcinoma (PDAC) resectability after neoadjuvant therapy (NAT) remains a diagnostic challenge. Traditional computed tomography (CT) criteria often fail to distinguish viable tumor from fibrosis, necessitating a reassessment of imaging-based standards. **Methods:** A comprehensive literature review was conducted using PubMed, focusing on prospective and retrospective studies over the past 25 years that evaluated the role of CT and complementary imaging modalities (MRI, PET-CT) in predicting resectability post-NAT in non-metastatic PDAC. Studies with small sample sizes or case reports were excluded. **Results:** Across studies, conventional CT parameters—particularly >180° vascular encasement—showed a limited correlation with histologic invasion or surgical outcomes after NAT. Persistent vessel contact on CT often reflected fibrosis, rather than active tumor. Dynamic changes, such as regression in the tumor–vessel interface and vessel lumen restoration, correlated more accurately with R0 resection. Adjunct markers like CA 19-9 response and patient performance status further improved resectability prediction. **Conclusions:** CT-based resectability assessment after NAT should transition from static morphologic criteria to response-based interpretation. Multidisciplinary evaluation integrating radiologic, biochemical, and clinical findings is essential to guide surgical decision-making and improve patient outcomes.

## 1. Introduction

Pancreatic ductal adenocarcinoma (PDAC) is a biologically aggressive malignancy with a rising global incidence and a persistently poor prognosis. It ranks among the top causes of cancer-related mortality, with a five-year survival rate remaining below 10% [1]. This unfavorable outcome is primarily attributed to the disease’s propensity for early systemic dissemination, perineural invasion, and the frequent encasement of major vascular structures, which collectively limit the number of patients eligible for curative surgical resection [2,3]. At diagnosis, most patients present with locally advanced or metastatic disease. For patients with localized tumors, surgical resection followed by adjuvant chemotherapy offers the only potentially curative option, but the achievement of negative surgical margins (R0 resection) is critical for long-term survival [4].

**Initial imaging and staging:** Contrast-enhanced multidetector CT is the standard-of-reference imaging modality for the initial staging of PDAC. Classification systems based on pancreatic protocol CT findings are used to categorize tumors as resectable, borderline resectable (BRPC), locally advanced (LAPC), or unresectable. This is primarily based on the tumor’s relationship to adjacent vasculature [5,6]. Resectable tumors exhibit no arterial or significant venous involvement, maintaining clear fat planes around the superior mesenteric artery (SMA), celiac axis, and portal vein (PV). Borderline resectable tumors typically exhibit limited vascular involvement—also referred to as vascular abutment—(≤180° circumferential contact with major arteries or short-segment venous narrowing) that may be compliant with vascular resection or reconstruction [7,8]. Locally advanced tumors are characterized by more extensive involvement (>180° encasement of critical arteries or occlusion of the superior mesenteric vein/portal vein confluence not reconstructible), which traditionally precludes immediate surgery. Tumors that present distant metastases or diffuse vascular encasement are considered unresectable [9,10].

**Pancreatic protocol CT technique:** High-quality multiphasic CT is crucial for optimal tumor detection and vascular assessment. A dedicated pancreatic CT protocol typically includes thin-section (≤3 mm) imaging with intravenous contrast during both the late arterial (pancreatic parenchymal) phase ~40–50 s after injection and the portal venous phase ~65–70 s, accompanied by multiplanar reconstructions for detailed evaluation [11]. This technique maximizes tumor imaging prominence—PDAC classically appears hypoenhancing relative to normal pancreas—and allows the accurate delineation of tumor–vessel interfaces and involvement of surrounding organs and structures. If initial CT images are not acquired in a pancreas protocol, it is recommended to repeat imaging with an appropriate technique in order to ensure precise staging. Utilizing proper imaging protocols does improve diagnostic accuracy and confidence in determining a tumor’s resectability [11].

**Neoadjuvant therapy (NAT) and the need to reassess resectability criteria:** The advent of NAT, including multiagent chemotherapy with or without radiation, has redefined the treatment plan for patients with BRPC and LAPC. NAT aims to downstage tumors, increase the likelihood of an R0 resection by sterilizing margins, eliminate micrometastatic disease, and allow only the biologically favorable tumors to proceed to surgery. Notably, there is growing evidence that NAT can convert initially unresectable or borderline tumors into candidates for curative resection, even in the presence of arterial involvement, which was once considered prohibitive. For example, surgical series have reported successful resections of tumors encasing the celiac axis or SMA following robust NAT responses, cases that historically would have been considered inoperable [6,12].

**Figure 1:**
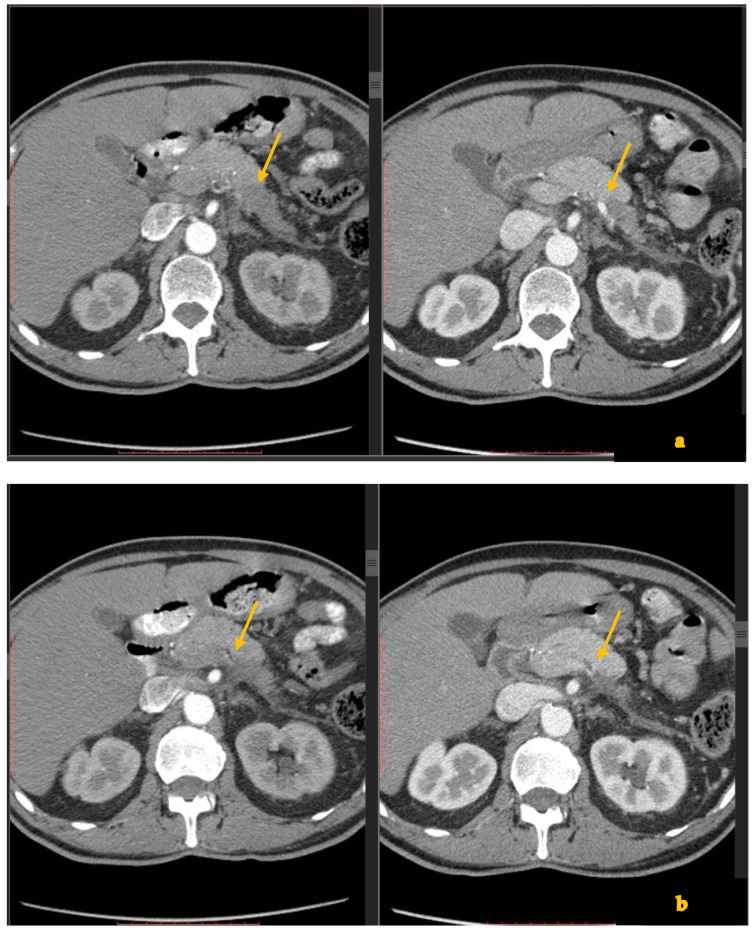
(**a**,**b**) Contrast-enhanced CT shows a large mass (arrows) in the pancreatic body and tail (**a**). After chemotherapy (**b**), there is improvement, but there is still visible tissue (arrows) close to the superior mesenteric artery, which was proven on surgery to represent fibrosis.

Despite these therapeutic advances, the radiologic assessment of treatment response and post-NAT resectability remains challenging. This is often due to therapy-induced fibrosis, necrosis, and inflammation, which can persist as mass-like tissue on imaging, obscuring the distinction between residual tumor and scar-desmoplastic reaction. Contrast-enhanced CT remains the primary imaging modality for restaging after NAT, but it has well-recognized limitations [13,14]. Persistent soft-tissue encasement of >180° around arteries or veins—a finding historically equated with unresectability—may represent fibrotic desmoplastic reaction, rather than viable tumor, post-NAT [15,16] (Figure 1).

Similarly, tumor size on CT may not change appreciably despite a major pathological response, often due to stromal collapse or therapy-related edema. Conventional radiographic criteria, such as absolute tumor diameter or static degrees of vessel contact, often fail to correlate with histopathologic response following treatment [17,18]. These limitations underscore the need for a redefined post-NAT imaging assessment that goes beyond pre-treatment anatomic criteria and incorporates both morphologic and dynamic features.

**Figure 2:**
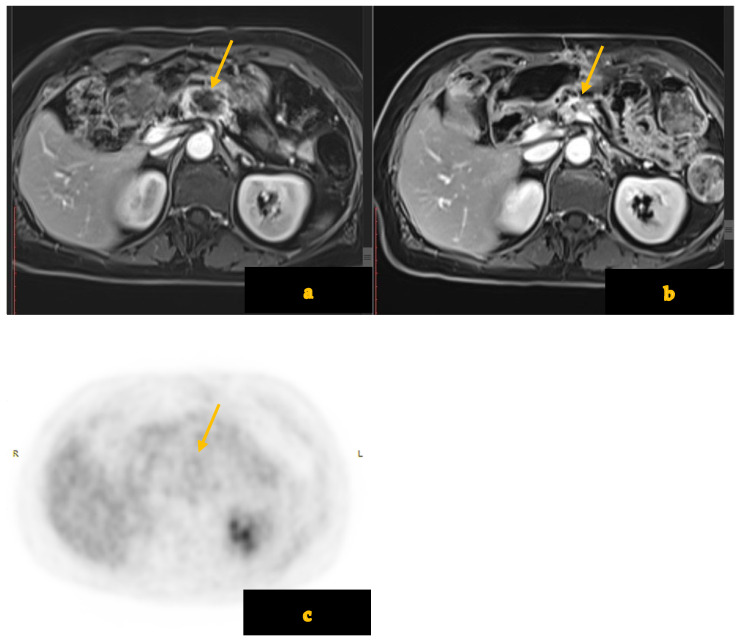
(**a**–**c**) Contrast-enhanced MRI shows a necrotic mass (arrow) in the pancreatic neck (**a**). After chemotherapy (**b**), there is a good response concerning the mass diameter and the vascular compression (arrow). FDG-PET (**c**) shows no uptake in the pancreas (arrow).

Moreover, complementary imaging modalities and techniques are being explored to improve post-therapy evaluation. Perfusion CT, diffusion-weighted MRI (DW-MRI), and PET/CT can provide functional insights into tumor biology (vascular perfusion, cellularity, and metabolic activity) and may aid in distinguishing fibrosis from residual tumor [19,20] (Figure 2).

For instance, early studies have shown that changes in perfusion parameters or ADC values on DW-MRI can correlate with treatment response [21,22]. PET with ^18FDG can demonstrate metabolic tumor response that can sometimes precede morphologic change [23]. However, these techniques lack standardized response criteria and can lead to variable results, so CT remains the cornerstone of assessment, often supplemented with such modalities [24]. Additionally, evolving surgical strategies—including planned vascular resections and reconstructions—have broadened the surgical candidacy of patients with residual vascular involvement following NAT [25,26,27].

**Objectives:** In light of these developments, a consensus on standardized imaging criteria for post-NAT resectability is lacking, so radiologists must often rely on their experience and multidisciplinary input. This review aims to synthesize current evidence and expert recommendations regarding CT evaluation of PDAC after neoadjuvant treatment. We highlight the efficacy and limitations of conventional CT criteria in this setting, examine emerging imaging signs of treatment response, and discuss how interpretation should evolve to guide surgical decision-making in the modern era of NAT.

## 2. Materials and Methods

We performed a comprehensive literature search to examine the evolving imaging criteria used to assess PDAC resectability following neoadjuvant therapy. The PubMed database was systematically queried for English-language articles published in the last 25 years focusing on post-NAT radiologic evaluation. Search terms included combinations of “pancreatic cancer,” “neoadjuvant therapy,” “CT,” “resectability,” “vascular involvement,” and “imaging response.” Both prospective and retrospective studies were included, as well as relevant meta-analyses and consensus guideline papers. Selection priority was given to studies directly evaluating the accuracy of CT (as well as other imaging modalities like MRI or PET) in predicting surgical resectability or pathologic response after NAT. Studies dealing with only pre-treatment imaging were excluded, as were small case series or single case reports to maintain robust evidence. In total, we identified several relevant studies and extracted data on imaging findings (tumor size, vessel involvement, etc.), their correlation with surgical/pathological outcomes, and any proposed new criteria or qualitative signs. These findings were synthesized qualitatively for this narrative review. No formal meta-analysis was performed, given the heterogeneity in study designs and endpoints, but consensus trends across the literature were emphasized.

## 3. Results

**Figure 3:**
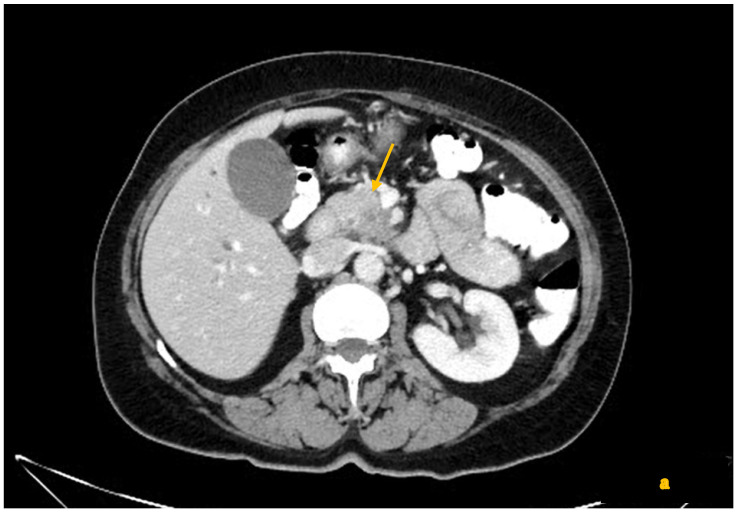

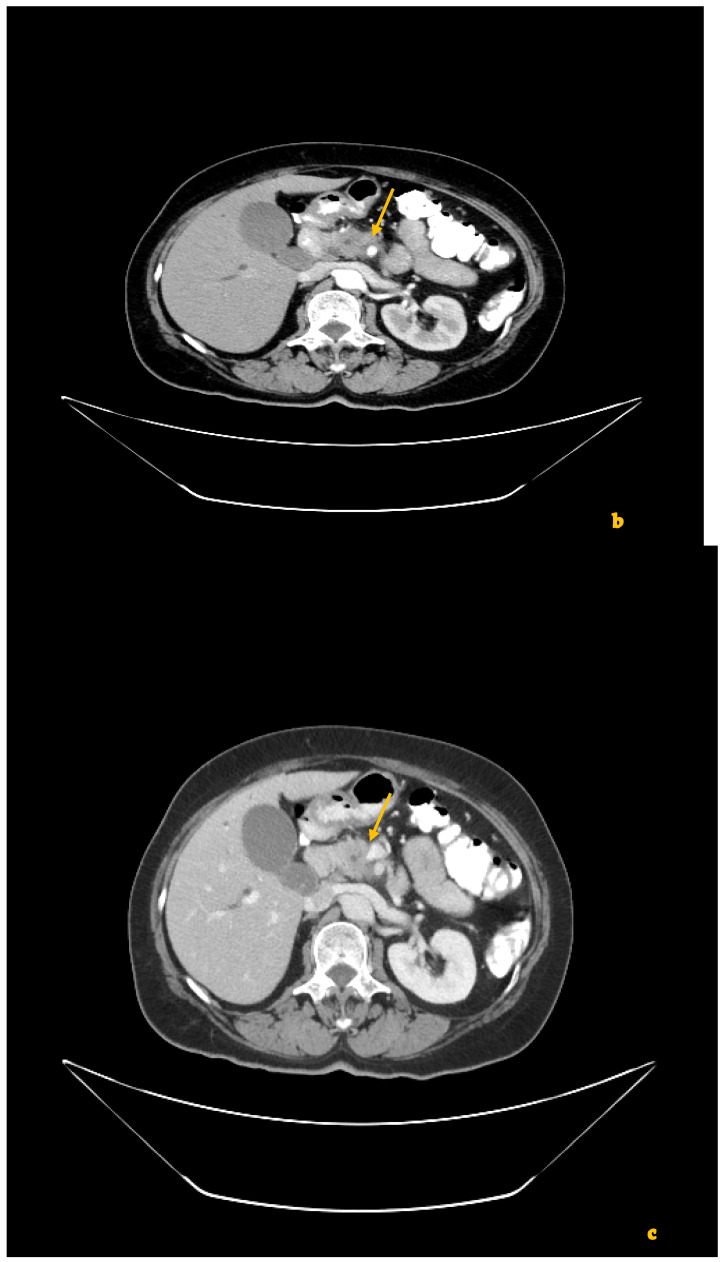
(**a**–**c**) Contrast-enhanced CT shows a locally advanced pancreatic cancer in the uncinate process (arrow), infiltrating the superior mesenteric vessels with mark deformation and retraction (**a**). After chemotherapy (**b**,**c**), there is still visible tissue close to the superior mesenteric vessels but to a lesser extent, specifically around the superior mesenteric artery (arrow) that seems no more retracted (**b**), while retraction persists in the superior mesenteric vein (arrow), probably due to fibrosis (**c**).

The evidence synthesized from multiple studies, including prospective trials, retrospective analyses, expert panel recommendations, and meta-analyses, reveals a paradigm shift in the radiologic assessment of PDAC resectability after neoadjuvant therapy. Collectively, these data demonstrate that traditional CT criteria—particularly tumor size and the extent of tumor–vessel contact—are insufficiently reliable for predicting surgical resectability or true residual tumor status in the post-NAT setting. A recurring finding is a marked reduction in the specificity of CT for detecting actual tumor invasion of vessels after NAT. CT frequently overestimates residual tumor involvement of vessels, likely due to the presence of fibrosis following therapy that maintains a mass effect or encasement appearance (Figure 3).

**Figure 4:**
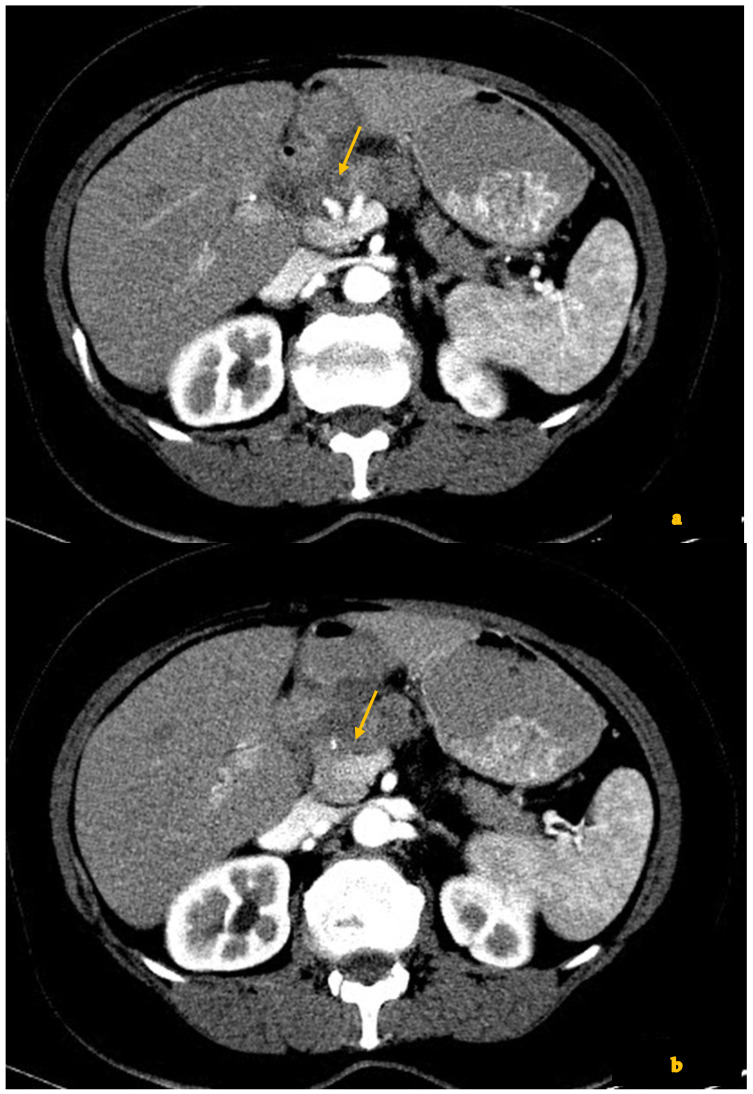

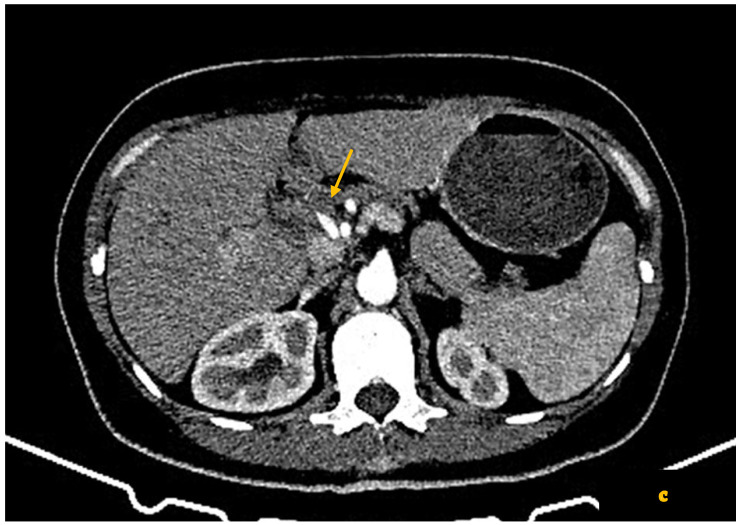
(**a**–**c**) Contrast-enhanced CT shows a locally advanced pancreatic cancer in the pancreatic neck (arrow) infiltrating the common hepatic artery and the splenic artery (**a**), as well as the portal vein (arrow) (**b**). After chemotherapy (**c**), the mass is smaller in diameter, and there is less extension to adjacent vessels (arrow). The patient underwent a complete R0 resection.

For instance, in a study by Cassinotto et al., the specificity of CT for R0 resectability dropped from ~88% before therapy to ~52% after NAT, with CT often overcalling vascular invasion that was not found at surgery. They noted that 12 of 31 patients who achieved complete (R0) resection after NAT had been interpreted as “high risk of incomplete resection” on restaging CT due to apparent vascular involvement [18]. Similarly, White et al. observed that restaging CT after chemoradiation tended to overstage disease, excluding some patients from surgery who actually could have undergone R0 resection [28]. These findings underscore that the use of static pre-treatment CT cutoffs (such as >180° arterial encasement) in the post-treatment context will often underestimate resectability. A particularly illustrative result comes from Mayer et al., who specifically evaluated arterial encasement >180° in post-NAT CT. They found that only approximately one-third of arterial segments that still appeared encased >180° on CT had actual histologic tumor invasion of the artery. In their series, many patients with persistent circumferential arterial contact on CT were nevertheless able to undergo successful R0 resections [29]. This suggests that a substantial proportion of what looks like artery “encasement” after therapy is, in fact, due to desmoplastic reaction or an inflammatory tissue, rather than viable cancer. The same appears true for veins: multiple studies reported that persistent vein narrowing or occlusion on CT after NAT does not always signify unresectability, as the vein can often be safely resected or even found patent at surgery with only fibrous adhesions remaining. For example, Cassinotto et al. noted that, even when the superior mesenteric vein (SMV) or portal vein remained stenosed in post-therapy CT, this finding was not predictive of an R1 resection—in their cohort, vein narrowing often did not equate to residual tumor at the margin. In contrast, when they did observe any partial regression of the tumor–vessel interface on CT (even if the vessel was still contacted but to a lesser degree or over a shorter length), it was a very positive sign: partial regression of tumor contact with the SMV/PV was associated with R0 resection in 100% of cases in that study. Partial regression of contact with any major vessel (arterial or venous) predicted R0 resection in 91% of cases [18]. These statistics highlight how changes in the tumor–vessel interface are more prognostically meaningful than the static presence of contact itself (Figure 4).

**Figure 5:**
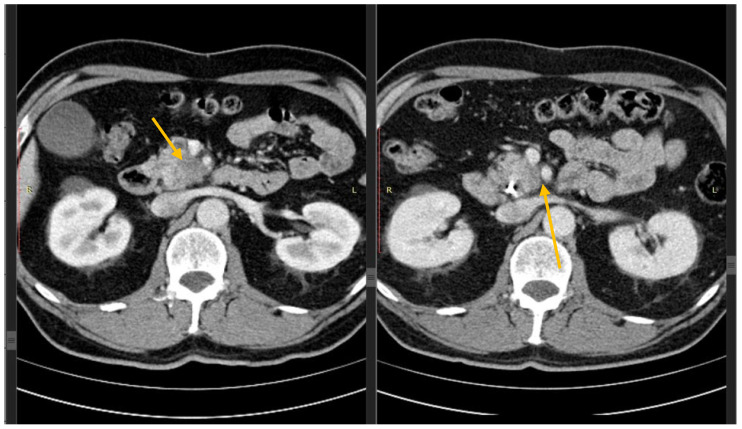
Contrast-enhanced CT shows on the left a hypovascular mass in the uncinate process (arrow), retracting the superior mesenteric vein and infiltrating the superior mesenteric artery. After chemotherapy (on the right), the mass is smaller, while a clearly visible fat plane is now seen between the lesion and the superior mesenteric artery (arrow), which represents the absolute criterion for resectability.

Another key observation across the literature is that qualitative changes in perivascular tissue and vessel patency on CT correlate better with outcomes than do gross tumor size changes. Zins et al. have emphasized that simple metrics like tumor diameter or even CT attenuation change have poor correlation with pathologic response or tumor resectability. Instead, radiologists are noting features such as the following: (a) a reduction in the circumferential degree of vessel contact, (b) the restoration of a normal vessel contour or lumen caliber, and (c) the development of a perivascular hypoattenuating “halo” replacing what was previously solid tumor around a vessel, as more indicative of a favorable response [16] (Figure 5).

The “halo sign” refers to the appearance of a low-density rim between the tumor and vessel inpost-NAT CT where previously the tumor was in direct contact—essentially an ill-defined fatty or scar tissue plane enveloping the vessel. Its appearance has been linked with tumor regression and the potential for a safe dissection plane surgically. In one series, the presence of a low-contrast perivascular halo (≤~46 Hounsfield units) adjacent to an artery was identified as the unique CT feature distinguishing cases where the artery was not truly invaded [30].

Importantly, even cases without vast radiologic tumor shrinkage can still be resectable. Several studies documented that objective RECIST partial responses on imaging are relatively uncommon with NAT (often, the majority of cases are radiologically “stable disease”), yet a substantial subset of these patients still achieve R0 resections and major pathologic tumor clearance. Marthey et al. and Xia et al. reported that, among borderline/locally advanced PDAC patients who completed NAT and went to surgery, only ~10–15% showed partial tumor shrinkage on CT, while ~70% had stable disease by size criteria. Despite this, R0 resection rates in these surgical patients were high (often >90% in responders), indicating that the lack of a radiologic tumor size reduction does not equate to the lack of a treatment effect [31,32]. Histopathologically, many of these “stable disease” tumors have extensive fibrosis or treatment-induced necrosis, explaining why they remained radiologically unchanged in size. The meta-analysis of Suker et al. noted that many patients with stable or minimal response on imaging following NAT with FOLFIRINOX still underwent successful resections [33].

Similar findings come from large meta-analyses and series. A pooled analysis by Gillen et al. found that NAT enabled surgical resection in roughly 26% of patients with initially unresectable or borderline tumors, with an overall R0 resection rate of ~77% among those resected [34]. More recent multi-institution studies (e.g., Ferrone et al.) report even higher conversion rates: in Ferrone’s series, ~30% of LAPC patients were ultimately resected after FOLFIRINOX, and remarkably, 92% of those who reached the operating table had an R0 resection. Notably, the majority (≈70%) of Ferrone’s patients still had tumors appearing “borderline or unresectable” in post-NAT CT—underscoring how imaging often did not reflect the true resectability that became evident intraoperatively. In their cohort, nearly half of the patients deemed unresectable by imaging after NAT were found to be resectable at surgery, with successful margin-negative outcomes in most [35]. These outcomes collectively reinforce the idea that persistent CT findings of vessel involvement after NAT, especially venous involvement, should not be viewed as an absolute contraindication to exploration. Instead, a comprehensive evaluation incorporating interval imaging changes (rather than static findings), tumor marker dynamics (e.g., CA 19-9 drop), and clinical status should guide the decision of whether to proceed to operation [24].

In summary, our review finds consistent evidence advocating for a shift away from rigid pre-NAT radiologic cutoffs toward a more response-based, multidimensional assessment after neoadjuvant therapy. Key imaging features correlating with resectability include any decrease in tumor–vessel contact extent, an improvement in vessel patency or contour (particularly venous re-canalization), and the appearance of fatty or hypoattenuating planes (halo) around vessels. Conversely, unchanged extensive encasement on CT is not always reliable—many such cases can still be R0 resections due to sterilized tumor tissue. Adjunctive factors like biochemical tumor markers and patient performance status further improve selection. Taken together, these findings support updated imaging criteria and reporting strategies that emphasize interval change over absolute thresholds, and they highlight the importance of multidisciplinary tumor board discussions in interpreting post-therapy imaging for PDAC.

## 4. Discussion

**Introduction to Post-Neoadjuvant Imaging Interpretation**

The evidence synthesized from multiple studies, including prospective trials, retrospective analyses, expert panel recommendations, and meta-analyses, reveals a paradigm shift in the radiologic assessment of PDAC resectability after neoadjuvant therapy (NAT). Traditional CT criteria—particularly tumor size and the extent of tumor–vessel contact—are increasingly unreliable in the post-NAT setting due to treatment-induced fibrosis and desmoplastic reactions that can mimic residual tumor.

**Tumor–Vessel Interface: Regression vs. Persistence**

Distinguishing viable tumor from fibrotic or necrotic tissue at the tumor–vessel interface is a key interpretative challenge. Studies have shown that post-NAT imaging frequently overestimates the tumor involvement of vessels. For instance, Cassinotto et al. found a drop in CT specificity for R0 resectability from ~88% pre-NAT to ~52% post-NAT due to false-positive interpretations of vascular invasion. Partial regression of tumor–vessel contact, such as decreased length or degree of encasement, is highly predictive of successful resection (R0), even when some contact persists. This has prompted a move toward assessing interval change in vessel involvement rather than relying on static cutoffs.

**Qualitative Imaging Signs: Halo Sign and Vessel Contour Restoration**

Qualitative changes in perivascular tissue, such as the appearance of a hypoattenuating “halo” between tumor and vessel, are emerging as important indicators of a favorable treatment response. The halo suggests the presence of scar or fat tissue, implying that tumor regression has occurred. This sign, when combined with restored vessel lumen and reduced contact area, has a high predictive value for safe surgical dissection and R0 resection [36,37].

**Discrepancy Between Tumor Size and Pathologic Response**

Objective tumor shrinkage (RECIST partial response) is relatively rare post-NAT, yet many patients still achieve R0 resections. Studies such as those by Marthey et al. and Xia et al. demonstrated high rates of successful resection in patients who had stable disease on imaging, emphasizing that radiologic size alone does not reflect biological response. These tumors often exhibit significant necrosis or fibrosis on histopathology.

**Arterial vs. Venous Involvement: Distinct Imaging Behaviors**

Post-NAT imaging interpretation must distinguish between arterial and venous responses [38]:

Arterial involvement: CT signs such as >180° encasement are no longer definitive for unresectability. Desmoplastic tissue may encase arteries without actual invasion. Studies suggest that partial or incomplete arterial contact post-NAT is often resectable, especially if accompanied by halo signs or preserved vessel contour.

Venous involvement: Veins (e.g., SMV, PV) frequently recanalize after NAT, and persistent occlusion may still be surgically manageable. The restoration of patency or improved caliber is a strong indicator of treatment response. Persistent narrowing alone is not predictive of R1 resections and should not preclude exploration.

**Optimal Timing for Imaging Assessment**

The optimal timing for imaging assessment after pancreatic cancer treatment depends on the treatment type and the specific goals of the imaging. Generally, imaging is performed 3–6 months after treatment to assess response and detect recurrence. However, for patients with resectable disease, imaging may be performed closer to the surgical date, while those undergoing neoadjuvant therapy may have imaging scans conducted more frequently to monitor treatment response. Patients should undergo a repeat MDCT within 25 days of any planned definitive operative intervention for pancreatic cancer to avoid unexpectedly finding metastatic disease at surgery.

**Role of Multidimensional Assessment**

Finally, it is important to emphasize the role of a multidisciplinary approach in evaluating resectability after NAT. A collaborative decision-making process that incorporates clinical findings, imaging results, laboratory data, patient comorbidities, and preferences is essential. This process typically involves input from surgeons, radiologists, oncologists, pathologists, and other relevant specialists to ensure a well-rounded and individualized treatment plan. Radiologic findings should be considered alongside serum tumor markers (e.g., a dramatic drop in CA 19-9 strengthens the argument that a radiologically static mass may be largely fibrotic) and the patient’s overall condition. Surgeons experienced in pancreatic cancer should be directly involved in imaging review; sometimes, a surgeon may judge that, even though a vessel looks involved, they have strategies to handle it, especially if other indicators (like lack of metastases and patient fitness) are favorable. The decision to proceed to surgery after NAT often comes down to whether there is any chance for R0 resection and no evidence of metastatic disease. Given the disconnect between radiographic appearance and pathologic reality in many cases, tumor boards increasingly err on the side of surgical exploration if imaging is equivocal but other factors are encouraging. This approach has led to improved outcomes for some patients who previously would have been deemed unresectable from imaging alone.

In summary, post-neoadjuvant imaging requires a paradigm shift in interpretation. Radiologists must embrace a more dynamic assessment, noting how tumor–vessel relationships have changed, rather than just whether they are present. Understanding the differences in arterial and venous responses and recognizing signs like the halo can provide critical guidance. However, imaging is one piece of the puzzle. Ongoing research into imaging biomarkers and the adoption of advanced analytics hold promise for closing the gap between radiographic assessment and the ground truth of tumor viability.

**Limitations of Imaging and Emerging Solutions**

Despite improvements in our understanding, post-NAT imaging faces inherent limitations. CT cannot reliably distinguish live tumor cells from fibrotic tissue on a microscopic level. Some patients will have residual microscopic cancer in areas that appear “clean” on CT, leading to positive margins or early recurrence despite favorable imaging. Conversely, CT can also underestimate response in some cases—for instance, a treated tumor may not shrink much but could be mostly necrotic; unless there are calcifications or obvious necrotic patterns, CT might label it stable disease. There is also interobserver variability in interpreting subtle post-treatment changes (e.g., what one radiologist calls a halo, another might call persistent tumor). Standardized reporting templates and criteria could help reduce this variability. In fact, the Society of Abdominal Radiology and American Pancreatic Association has advocated for structured reporting that includes descriptions of tumor–vessel interfaces, degree of contact, and any interval changes or treatment effect signs [38]. Incorporating specific descriptors like “perivascular halo present/absent” or “vessel deformity resolved” can make reports more actionable in multidisciplinary discussions [39].

**Radiomics and Artificial Intelligence: Promise and Precautions**

An emerging tool to augment human assessment is radiomics and artificial intelligence. Radiomics involves extracting high-dimensional quantitative features from imaging that might capture subtleties of texture, attenuation distribution, or shape changes that are imperceptible to the naked eye. Preliminary studies are promising: for example, Wang et al. demonstrated that changes in certain radiomic features between pre- and post-NAT CT (so-called “delta radiomics”) were able to predict margin status and even survival, outperforming some conventional measures. In their study, a radiomic signature could identify which patients were likely to achieve an R0 resection after NAT and had improved overall survival [40]. Such tools, once validated, could be integrated into clinical workflows to provide an objective second opinion on whether residual tissue is likely a tumor or not. Similarly, machine learning algorithms might eventually analyze the patterns of enhancement and vessel changes to output a probability of pathological response.

An important consideration in the clinical application of artificial intelligence (AI) is the issue of safety, particularly due to the “black box” nature of many AI models. These models, especially deep learning-based systems, often lack transparency in how decisions are made, which can pose risks in high-stakes environments such as healthcare. To mitigate these risks, it is essential to employ interpretable AI models where possible, ensure robust validation across diverse patient populations, and maintain human oversight in the decision-making process. In the context of pancreatic cancer resectability, recent studies have begun integrating explainable AI (XAI) techniques—such as Local Interpretable Model-agnostic Explanations (LIME) and Shapley Additive Explanations (SHAP)—to clarify model predictions and enhance clinician trust. Additionally, regulatory frameworks and guidelines are needed to govern the safe integration of AI tools into clinical workflows [41,42,43,44].

**Advanced Imaging Techniques**

Additional modalities like dual-energy CT (DECT), perfusion CT, diffusion MRI (ADC mapping), and PET/MRI hybrids are under investigation. These techniques show potential for differentiating a viable tumor from fibrosis but require further validation before routine clinical adoption [45].

## 5. Conclusions

Neoadjuvant therapy has introduced new complexities to the imaging evaluation of pancreatic cancer, and it is clear that traditional CT resectability criteria should not be applied uncritically in the post-therapy setting. Persistent tumor–vessel contact or even encasement on CT after NAT does not automatically equate to unresectability, as these findings frequently reflect scar tissue, rather than active cancer. Evidence from recent studies shows that paying attention to treatment-induced changes—such as a decrease in vessel’s involvement, the restoration of venous caliber, or the appearance of a low-attenuation perivascular rim—can substantially improve the accuracy of radiologic resectability predictions. Conversely, static features like tumor size or original degree of encasement alone are poor predictors of pathological response and outcome.

Accordingly, we advocate for a transition from rigid morphologic criteria to a more nuanced, response-based interpretation of post-NAT imaging. Radiologists should explicitly compare pre- and post-therapy scans to identify subtle signs of regression or progression, rather than focusing only on absolute post-treatment findings. Our review underscores the value of multidisciplinary evaluation: imaging results should be integrated with biochemical markers (e.g., CA 19-9 trends), clinical status, and surgical judgment. In practice, this means that borderzone cases on imaging are often best managed through collective discussion. For example, a patient with a stable disease according to CT but a major CA 19-9 drop and good performance status may justifiably be offered surgical exploration, whereas one with concerning clinical deterioration or rising biomarkers might be redirected from surgery, even if imaging is unchanged.

Looking forward, the refinement of imaging criteria will likely involve both qualitative improvements (better recognition of signs like the halo, string, etc.) and quantitative tools (radiomic algorithms and standardized scoring systems). Such advancements could improve our ability to stratify patients—identifying those for whom surgery will truly be beneficial and sparing those who are unlikely to benefit from the morbidity of an operation. In the meantime, adopting standardized reporting templates that highlight key post-NAT findings can aid communication and decision-making.

In conclusion, assessing PDAC resectability after neoadjuvant therapy requires a departure from the classical one-dimensional criteria. By embracing a comprehensive approach that accounts for treatment-related changes on CT and validating evidence from other modalities, clinicians can better select patients for surgery. This strategy offers the dual benefit of improving oncologic outcomes (by offering surgery to those who can achieve R0 resections) and avoiding futile laparotomies in those unlikely to benefit. As therapies improve and more patients receive NAT, the radiologist’s role in guiding management will only grow—making the continual reassessment of our imaging criteria not only prudent but also necessary for optimizing care in pancreatic cancer.

Summary: vascular contact and tumor encasement seen in post-treatment CT do not necessarily mean unresectability.

Imaging findings should be integrated with clinical status and biochemical markers. The essential points for evaluating resectability are as follows:

- Changes in the tumor–vascular interface.
- Low-attenuation halo sign.
- A decrease in CA 19-9 levels.

## Data Availability

The data presented in this study are available upon request from the corresponding author.
